# Machine Learning for Diagnosis of Hematologic Diseases in Magnetic Resonance Imaging of Lumbar Spines

**DOI:** 10.1038/s41598-019-42579-y

**Published:** 2019-04-15

**Authors:** Eo-Jin Hwang, Joon-Yong Jung, Seul Ki Lee, Sung-Eun Lee, Won-Hee Jee

**Affiliations:** 1 Department of Radiology, Seoul St. Mary’s Hospital, College of Medicine, The Catholic University of Korea, Seoul, Republic of Korea; 2 Department of Hematology, Seoul St. Mary’s Hospital, College of Medicine, The Catholic University of Korea, Seoul, Republic of Korea

## Abstract

We aimed to assess feasibility of a support vector machine (SVM) texture classifier to discriminate pathologic infiltration patterns from the normal bone marrows in MRI. This retrospective study included 467 cases, which were split into a training (n = 360) and a test set (n = 107). A sagittal T1-weighted lumbar spinal MR image was normalized by an intervertebral disk, and bone marrows were segmented. The various kernel functions and SVM input dimensions were experimented to construct the most optimal classifier model. The accuracy and sensitivity increased as the number of training set sizes increased from 180 to 360. The test set was analyzed by SVM and two independent readers, and the accuracy and sensitivity of the SVM classifier, reader 1 and reader 2 were 82.2% and 85.5%, 79.4% and 82.3%, and 82.2% and 83.9%, respectively. The area under receiver operating characteristic curve (AUC) of the SVM classifier, reader 1 and reader 2 were 0.895, 0.879 and 0.880, respectively. The SVM texture classifier produced comparable performance to radiologists in isolating the hematologic diseases, which could support inexperienced physicians with spinal MRI to screen patients with marrow diseases, who need further diagnostic work-ups to make final decisions.

## Introduction

Magnetic resonance imaging (MRI) of the spine is frequently performed in patients with low back pain because it directly visualizes vertebral column, spinal cords and nerves, and supporting soft tissue structures^1^. Bone marrow is an important part to be interpreted in reading the spinal MRI. However, diffuse bone marrow infiltration may appear as normal due to its generalized and repetitive pattern involving the entire marrow spaces^2^. In addition, age-dependent variabilities and marrow reconversion in response to physiological oxygen demands complicate the bone marrow interpretation^3,4^. Therefore, it is often challenging for physicians to make decisions with bone marrow signals on MRI and to determine whether to proceed with further clinical and laboratory work ups.

A number of machine-learning based and automated imaging diagnoses and classification of diseases have been studied in the field of medical imaging^5–8^. From a methodological point of view, features extracted from texture were used as inputs to the machine learning algorithm to build a decision-making model^6–8^. Texture analysis provides quantitative means to describe tissue properties and pathological stages to reveal information that is often invisible to the human eyes^9^. Based on the diffuse and redundant nature of the bone marrow infiltration on MRI, diffuse bone marrow diseases are propitious candidates for texture analysis. A previous study demonstrated the feasibility of texture analysis in determining the treatment response to the multiple myeloma^10^. Another recent study attempted to differentiate normal from abnormal marrows in metastases patients using a machine learning algorithm with textural inputs^11^. However, no attempt has been made to distinguish between normal and diffuse marrow infiltrative diseases using textural differences. We hypothesized that the machine-learning based algorithm with bone marrow textures as input would be able to discriminate the diffuse bone marrow infiltration from the normal bone marrows. Previously, a number of studies in the field of artificial intelligence had utilized support vector machine (SVM) as a classifier to differentiate non-medical images with various textures^12–14^. Therefore, the purpose of our study was to construct a machine learning based algorithm using a SVM texture classifier and to isolate infiltration patterns suspicious of hematologic diseases on lumbar spine MRI (L-spine MRI). We built a SVM texture classifier model that was most suitable for marrow differentiation and compared its performance to experienced radiologists on the separate data set. In addition, we estimated a sample size required to reach target classification accuracy.

## Results

### Effects of SVM kernel types and feature dimensions on predictive performance

**Figure 1:**
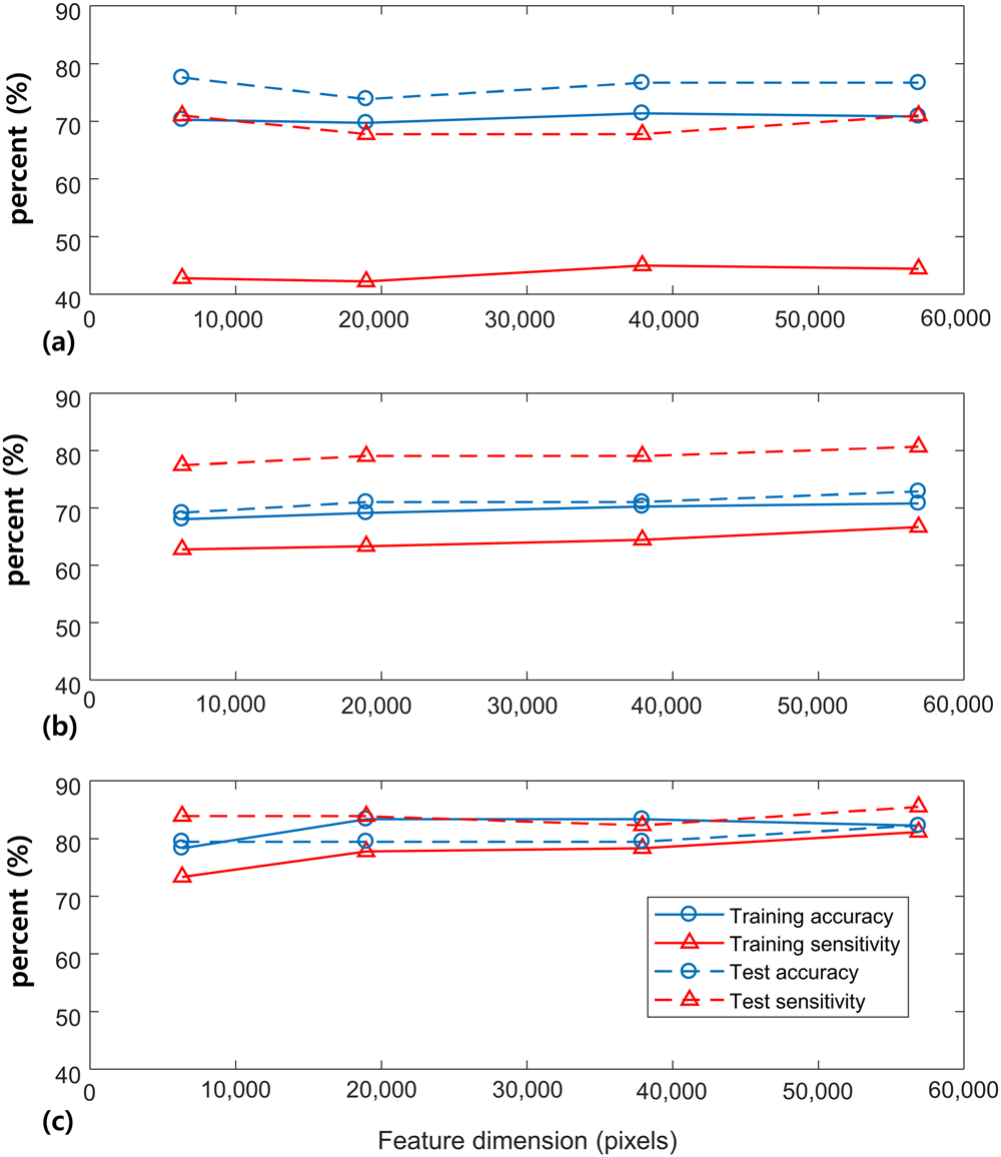
Training accuracy, test accuracy, training sensitivity and test sensitivity results experimented with different features dimensions and kernels at the given training set of 180 samples: (**a**) The 3^rd^ order polynomial kernel, (**b**) tangent hyperbolic kernel and (**c**) radial basis function kernel.

Figure 1 illustrates the effect of SVM kernels and feature dimensions on differentiating diseased marrows from the normal marrows. Overall, the classification accuracy and sensitivity were more affected by the choice of kernel types than they were on feature dimensions. The 3^rd^ order polynomial kernel produced the classification accuracy and sensitivity at the range of 60 to 80 percent depending on the feature dimension sizes. However, the training sensitivity only ranged from 40 to 50 percent (Fig. 1a). The tangent hyperbolic kernel produced varying accuracies and sensitivities between 60 to 80 percent, and the values did not increase linearly with the feature dimension sizes (Fig. 1b). The radial basis function kernel produced the accuracies and sensitivities at the range of 70 to 90 percent (Fig. 1c). The effect of feature dimension was minimal, and the results were consistent across all feature dimensions we tested. However, the feature dimension of 56,862 pixels produced the highest training sensitivity, and the difference between the training and test set results was minimal. We chose the radial basis function kernel and the feature dimension of 56,862 pixels to build the final model.

### Predictive performance of SVM on differentiating diseased marrows from the normal marrows

Table 1 illustrates the overall predictive performance of our SVM classifier model with respect to increasing training set sizes. Overall, the predictive performance of the marrow differentiation gradually increased with respect to the number of training set sizes. When the training set size was 360, the classification accuracy, sensitivity and specificity of the training sets and were 82.8%, 81.7%, 83.9%, respectively, and AUC was 0.895 (P < 0.001).

**Table 1:** The two-class SVM results with varying sizes of the training sets.

Number of training set		CA (%)	SE (%)	SP (%)	AUC[95% CI]
180	Highest	85.0	81.8	92.7	0.910[0.853–0.964]^*^
	Lowest	72.2	57.4	74.1	0.829[0.753–0.905]^*^
	Average	78.3(3.06)	71.7(6.51)	84.8(4.26)	0.867(0.0215)
240	Highest	87.1	82.1	93.7	0.925[0.874–0.975]^*^
	Lowest	76.3	66.9	75.5	0.848[0.776–0.920]^*^
	Average	80.5(2.37)	75.6(3.81)	85.3(3.51)	0.891(0.0190)
300	Highest	84.0	82.1	90.8	0.912[0.856–0.966]^*^
	Lowest	78.7	68.0	81.5	0.865[0.797–0.933]^*^
	Average	81.8(1.53)	78.3(2.83)	85.4(2.26)	0.890(0.0101)

Abbreviations: CA = classification accuracy SE = sensitivity, SP = specificity, AUC = area under the receiver operating characteristic curve, CI = Confidence Interval, *P < 0.001; Average outcomes from the 30 trials are presented with standard deviations in parentheses; The radial basis function kernel and feature dimension of 56,862 pixels were used for each number of training set.

### Comparison of performances and interobserver agreements between the SVM classifier and human readers using a separate data set

**Figure 2:**
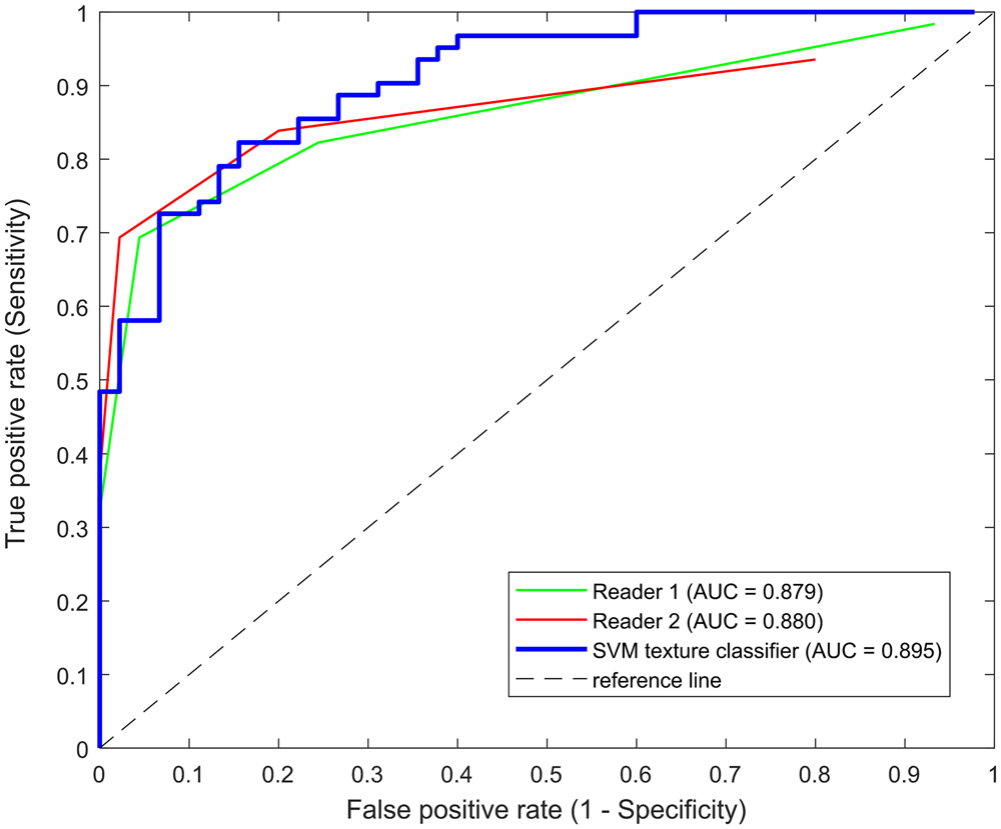
ROC curves showing the performances of two radiologists and SVM texture classifier in differentiating diseased bone marrows from the normal marrows. AUC of reader 1 was 0.879 [95% Confidence Interval (CI): 0.815–0.943; P < 0.001], of reader 2 was 0.880 [95% CI: 0.816–0.944; P < 0.001], and that of the SVM texture classifier was 0.895 [95% CI: 0.835–0.954; P < 0.001]. There was no significant difference between reader 1 and SVM (P = 0.282), or reader 2 and SVM (P = 0.392).

Table 2 illustrates the classification accuracy of SVM classifier and two independent readers for the same test set. There was no significant difference in accuracy, sensitivity and specificity between SVM and each reader. Figure 2 illustrates the ROC curves and AUCs of the SVM classifier, and two independent readers, respectively. There was no significant difference in AUCs between SVM and each reader.

**Table 2:** Classification accuracy, sensitivity and specificity of SVM, Reader 1 and Reader 2.

	CA (%)	SE (%)	SP (%)
SVM	82.2 [73.9–88.3]	85.5 [74.6–92.2]	77.8 [63.7–87.5]
Reader 1	79.4 [70.1–86.0], P = 0.999	82.3 [71.0–89.8], P = 0.790	75.6 [61.3–85.8], P = 0.803
Reader 2	82.2 [73.9–88.3], P = 0.999	83.9 [72.8–91.0], P = 0.999	80.0 [66.2–89.1], P = 0.999

Abbreviations: CA = classification accuracy SE = sensitivity, SP = specificity; Confidence interval are presented in brackets; P = p-value by McNemar tests for difference between each reader and SVM classifier; P < 0.05 was considered significant.

Interobserver agreements between SVM and readers were moderate: *κ* = 0.425 with reader 1 and *κ* = 0.599 with reader 2. This was similar to interobserver agreements between the two readers (*κ* = 0.560). Benign marrow signal changes involving at least one level of vertebra were vertebral hemangioma (n = 19), Modic type change (n = 37), and fracture (n = 30). A multivariate analysis revealed that the factor associated with SVM classification results was fracture (P = 0.018), but not hemangioma (P = 0.283) or Modic type change (P = 0.872). Nine false positive and false negative cases occurred by the SVM classifier in the test phase. Among them, the five false positive cases exhibited diffusely and heterogeneously decreased bone marrow signal intensities, which may be regarded as red marrow hyperplasia. Moreover, 6 false negative cases were multiple myeloma with normal marrow patterns.

### Predicting sample size required for classification

**Figure 3:**
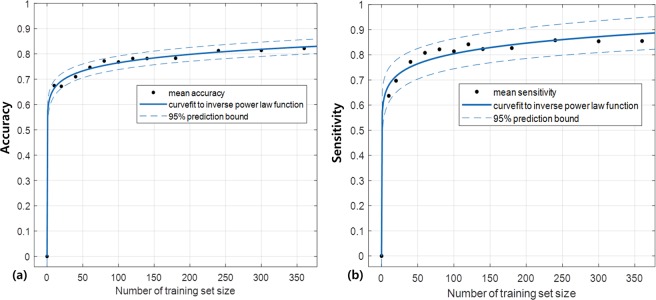
(**a**) The mean classification accuracies and (**b**) sensitivities with respect to increasing number of training sets. The fitted curves to the inverse power law function and the 95% prediction bounds are illustrated.

Figure 3 illustrates the change of classification accuracy and sensitivity with respect to the number of training samples and fitted curves to the inverse power law function. With the three coefficients calculated by curve-fit, it was estimated that the training samples more than 553 would be required to reach classification accuracy of 85% (Fig. 3a) and sensitivity of 91.4% (Fig. 3b). The SSE and RMSE values for predicting classification accuracy were close to zero, which indicated good fits to the learning curves. The three coefficients calculated by non-linear regression to the learning curve model and goodness-of-fit evaluation results are summarized in Supplementary Table S1.

## Discussion

We constructed a SVM texture classifier model to discriminate diffuse infiltrative patterns suspicious of hematologic diseases, which require further clinical and laboratory work-up to make a final decision. Its predictive performance was comparable to experienced radiologists, which successfully demonstrated the feasibility of SVM texture classifiers to differentiate bone marrow with hematologic diseases from those without diseases. The use of intervertebral disks or skeletal muscle signals in T1-weighted images has been internal standards in clinical practice since the initial study reported that they have helped determine pathologic marrow infiltrations with high accuracy^15^. However, the challenge for diagnosing marrow infiltrative diseases has been attributed to the presence of an overlap between normal hypercellular marrow and diffuse marrow infiltrations^16^. Moreover, a considerable proportion of multiple myeloma and the most of MGUS have been deluded as normal marrow patterns^17,18^. The majority of the falsely interpreted cases by the SVM classifier also falls under these challenges probably because the marrow signal was normalized by intervertebral disks. On the other hand, benign focal marrow lesions, such as vertebral hemangioma and Modic type changes, were not associated with the false interpretations. The association between fractures and SVM classifier was probably due to the fact that fracture is closely related to multiple myeloma in the diseased group^16,17^. The learning curve model predicted that both accuracy and sensitivity would increase with more number of training samples. Therefore, the future study would be needed to examine whether the accuracy would improve further with a larger sample size and outperform human observers.

We experimented with various user-defined parameters to find the most optimal SVM classifier model. The effects of different kernel types and input dimensions were evaluated and the combination of a kernel and input dimension with the best performance was determined. The SVM classifier searches for an optimal separating hyperplane that maximizes the margin of the nearest data points. These subsets of the data points are called support vectors (SVs), which fall closest to the separating hyperplane. The operation of SVM for texture classification is two-fold; nonlinear mapping of a texture space into a high-dimensional feature space and construction of a separating hyperplane in the feature space. For nonlinear mapping, a kernel function is used to map a textural input space into a high dimensional feature space^13^. In this study, three different types of kernels were experimented to find which one was most suitable for marrow differentiation, including the 3^rd^ order polynomial kernel, tangent hyperbolic kernel and radial basis function kernel. Among the three kernels, the radial basis function kernel produced the highest accuracy and sensitivity. The radial basis function kernel projects the data into infinite dimensions to find a linear separation^19^. Unlike the two other kernels, it builds a non-parametric model, which means the complexity of the model can grow infinitely with the size of the data. If one has the unlimited data and very weak prior knowledge about the data, the non-parametric model is always better than the parametric model, which makes the radial basis function kernel a popular and a good default kernel for SVM^20^.

For constructing a separate hyperplane in the features space, SVM is capable of using nonlinearly mapped input textures as features for classification. The textural input image is decomposed into a set of feature images using a bank of filters before classification is performed^12^. A multiple number of channels corresponding to different filters are necessary to capture specific characteristics of the input textures, which makes filter selection a major issue to discriminate appropriate textural properties. In high-dimensional feature space, SVM searches for SVs, which plays a role of filters that capture critical measures from the input image, which are identified by the operation performed by a kernel function^12^. Because SVM implicitly involves a process equivalent to feature selection, no additional feature selection was necessary. Therefore, we provided the gray-level pixels from the bone marrow images to SVM without additional user-defined feature selection methods. In general, a texture study follows the sequential steps of post-processing, feature extraction, feature selection, and classification^21^. A classification model confronts a risk of overfitting if too many features are included^22^, which makes feature selection an essential step in building a classification model^6,23^. In this study, we avoided the issues with feature selection by preserving the textural information from the data as itself. Moreover, avoiding features selection substantially reduced time and effort to build the final model.

Since the raw pixels from the images were directly used as inputs to SVM, no spatial smoothing or further filtering process was involved in our study. Non-medical image classification using SVM had required additional modelling or filtering, such as a multiresolution simultaneous autoregressive model^24^ or wavelet transform^25,26^. In fact, none of these methods improved the overall accuracy, which could be attributed to irregular patterns of the bone marrow textures. In addition, our method was designed to accept heterogeneity of the T1-weighted images from multiple MR machines and acquisition parameters. Each scanner might involve its own and distinctive internal filtering operation before the final image is generated, but we were unable to control these vender-specific filters, nor did the internally smoothed images seem to affect much on our results. Although the images from a single vendor and a single scan protocol may have produced better results with less number of sample sizes, we tried to minimize the discrepancy by adjusting the signal intensity levels with respect to the disk signal of the same subject.

The limitations were the following. First, there was a bias on our population in that a major proportion of our study population was multiple myeloma among other hematologic diseases, and the cases with hypercellular marrow secondary to anemia, which is relatively frequent in clinical practice, were not included. However, these hematologic diseases share similar imaging features in T1-weighted images because the marrow signal intensity is determined by the relative composition of cellular and fat components. Our main objective was to diagnose bone marrow infiltration, not to distinguish each category separately. Therefore, this biased population might not have influenced on the overall results. Second, we sampled the disease positive and negative data from the separate cohorts in a case-control manner. This sampling pattern carries a risk of biases that the samples might not adequately reflect the spectrum in real clinical practice^27^. Third, we did not consider demographic parameters. Age and gender are well-established factors accounting for heterogeneous signal intensities in the normal bone marrows^3,28^. In particular, exclusion of females aged less than 40 and males less than 30 due to the concerns for hypercellular marrows may narrow the applicability. In the future study, a model regarding demographic factors should also be considered. Fourth, external validation was not performed. We split the data for training and validation of the SVM classifier model. However, external validation using geographically different data set is preferred to ensure generalizability^27^. A method to control balance between false positives and false negatives should also be incorporated to increase and stabilize the sensitivity values^29^. Finally, both accuracy and sensitivity could be improved by including multi-modal images as inputs to SVM classifier such as short T1 inversion recovery (STIR) images, dynamic contrast-enhanced images, chemical shift images and diffusion-weighted images, which are frequently used to help determine marrow disease status in conjunction with the T1-weighted images^30–32^.

In summary, we introduced a machine learning method to differentiate diffuse marrow infiltrative diseases from the normal bone marrows based on the L-spine MRI. The SVM texture classifier model demonstrated comparable performance to experienced radiologists in isolating the marrows with hematologic diseases from the normal ones. In this respect, the SVM texture classifier has the potential to support physicians to determine whether the bone marrow signals suspicious of hematologic diseases would require further diagnostic work-ups.

## Methods

### Subjects

**Figure 4:**
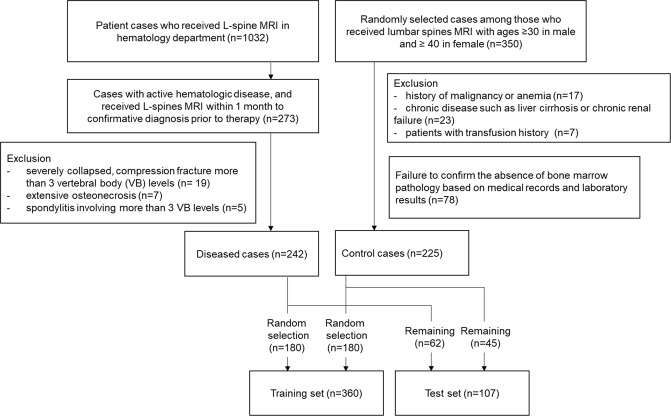
Flowchart demonstrating how the diseased and control groups were selected and assigned to the training or test sets. The diseased and control cases were collected from the separate cohorts in a retrospective case-control manner with different selection criteria, which were entirely based on the clinicopathological features without considering MR findings.

This retrospective study was approved by the institutional review board, and informed consent was waived. Figure 4 illustrates the flowchart of the subject inclusion criteria. The diseased and control cases were collected from the separate cohorts with different selection criteria, which were entirely based on the clinicopathological features without considering MR findings. For the diseased group, patients who visited the hematology department of our hospital and received L-spine MRI between March 2010 and June 2017 were searched in our PACS system (n = 1032). Among them, included were 273 patient cases from 256 patients (17 patients received MRI twice, one at the initial diagnosis and the other at relapse after complete remission) who met the following criteria: (1) confirmative diagnosis of having active hematologic disease based on the clinicopathological criteria of each disease category, (2) prior to initiation of the therapy in the first diagnosed patient, or re-initiation of the therapy in the relapsed patients. After reviewing 273 images, 31 were additionally excluded because bone marrow in their images were not appropriate for texture input due to combined diseases: severely collap sed, multilevel (>3 vertebral body segments) compression fracture (n = 19), extensive osteonecrosis (n = 7), spondylitis involving multiple levels (>3 vertebral body segments) (n = 5). The diseased group consisted of multiple myeloma (n = 159), leukemia (n = 32), lymphoma (n = 28), monoclonal gammopathy of unknown significance (MGUS) (n = 12), myelodysplastic syndrome (MDS) (n = 7), myelofibrosis (n = 3) and hypereosinophilia (n = 1).

For the control group, 350 cases were randomly selected from our PACS system among those who received L-spine MRIs between March 2010 and June 2017. Marrow cellularity usually depends on age and gender^33^. It has been known that young men and middle-aged women have less than 50% of fat components on average^34^. Therefore, the majority of people in this age group exhibited low bone marrow signal intensities on the T1-weighted MRI, which frequently leads to inconclusive interpretations. Because the aim of this study was to separate abnormal bone marrow signal intensities solely based on the texture information from MR imaging, females aged less than 40 and males less than 30 were excluded. Furthermore, the cases with history of malignancy or anemia, chronic diseases such as liver cirrhosis or chronic renal failure, and patients with transfusion history were excluded. The medical records and laboratory results longitudinally followed up for more than one year to confirm the absence of bone marrow pathology. Finally, 242 diseased cases (mean age: 60.3 ± 11.59, male: female = 131:111), and 225 control cases (mean age: 65.3 ± 12.41, male: female = 90:135) were included in this study. Among them, 180 cases were randomly selected for a training set from the diseased and control groups, respectively. The remaining cases were denoted as a test set. Consequently, 360 patients were assigned as a training group (mean age: 62.7 ± 11.76, male: female = 201:159, control: disease = 180:180), and 107 patients as a test group (mean age: 62.8 ± 13.77, male: female = 45:62, control: disease = 45:62).

### MRI acquisition

MR images were acquired using multiple MRI vendors (Table 3). The T1-weighted images used in our study were heterogeneous in terms of manufacturers, model names, magnetic fields and scanning parameters.

**Table 3:** Summary of the sequence parameters for multiple MR vendors.

	MR1	MR2	MR3	MR4	MR5	MR6	MR7
Manufacturer	Philips	Siemens	Siemens	Siemens	Philips	Siemens	GE
Model name	Ingenia	Verio	Verio	Verio	Achieva	Avanto	Signa HDxt
Scanning sequence	2D T1 TSE	2D T1 TSE	2D T1 TSE	2D T1 TSE	2D T1 TSE	2D T1 TSE	2D T1 FSE
Magnetic field (Tesla)	3	3	3	3	1.5	1.5	1.5
TR (msec)	700	700	797	750	531.1	450	585.3
TE (msec)	10	10	10	10	20	10	21.36
Flip angle (°)	90	128	128	128	90	150	90
Echo train length	5	3	3	3	5	3	3
Resolution (mm^2^)	0.59 × 0.59	0.55 × 0.55	0.625 × 0.625	0.55 × 0.55	0.55 × 0.55	0.67 × 0.67	0.51 × 0.51
Slice Thickness (mm)	3	3	3	3	3	3	3
Gap (mm)	3	3	3	3	3	3.3	3
Matrix size	512 × 512	512 × 512	448 × 448	512 × 512	512 × 512	448 × 448	512 × 512
Number of slices	21	19	19	23	19	19	19
Bandwidth (Hz/pixel)	291	250	250	250	154.8	172	108.5
Number of averages	1	1	1	1	3	2	0.5

### Image post-processing

All algorithms for post-processing were written and executed using a MATLAB software package (MATLAB and Statistics Toolbox 2017a, The Mathwork, Inc, Natick, MA, USA). To compensate for signal heterogeneities, the acquired T1-weighted images were normalized by subtracting the whole pixels from the annulus fibrosus of nondegenerated intervertebral disk of the same subject. The intervertebral disk was separated into 5 regions with equal distance from anterior to posterior, and the first and last regions were regarded as annulus fibrosus. The disk-normalized marrows were segmented using a 3-dimensional GrowCut algorithm, which is a semi-automatic algorithm to segment the area of interest from multiple slices of an image^35^. The sagittal T1-weighted images from multiple vendors and the processed images of the normal controls and patients with hematologic diseases can be found as Supplementary Fig. S1.

### Data preparation for SVM model construction

**Figure 5:**
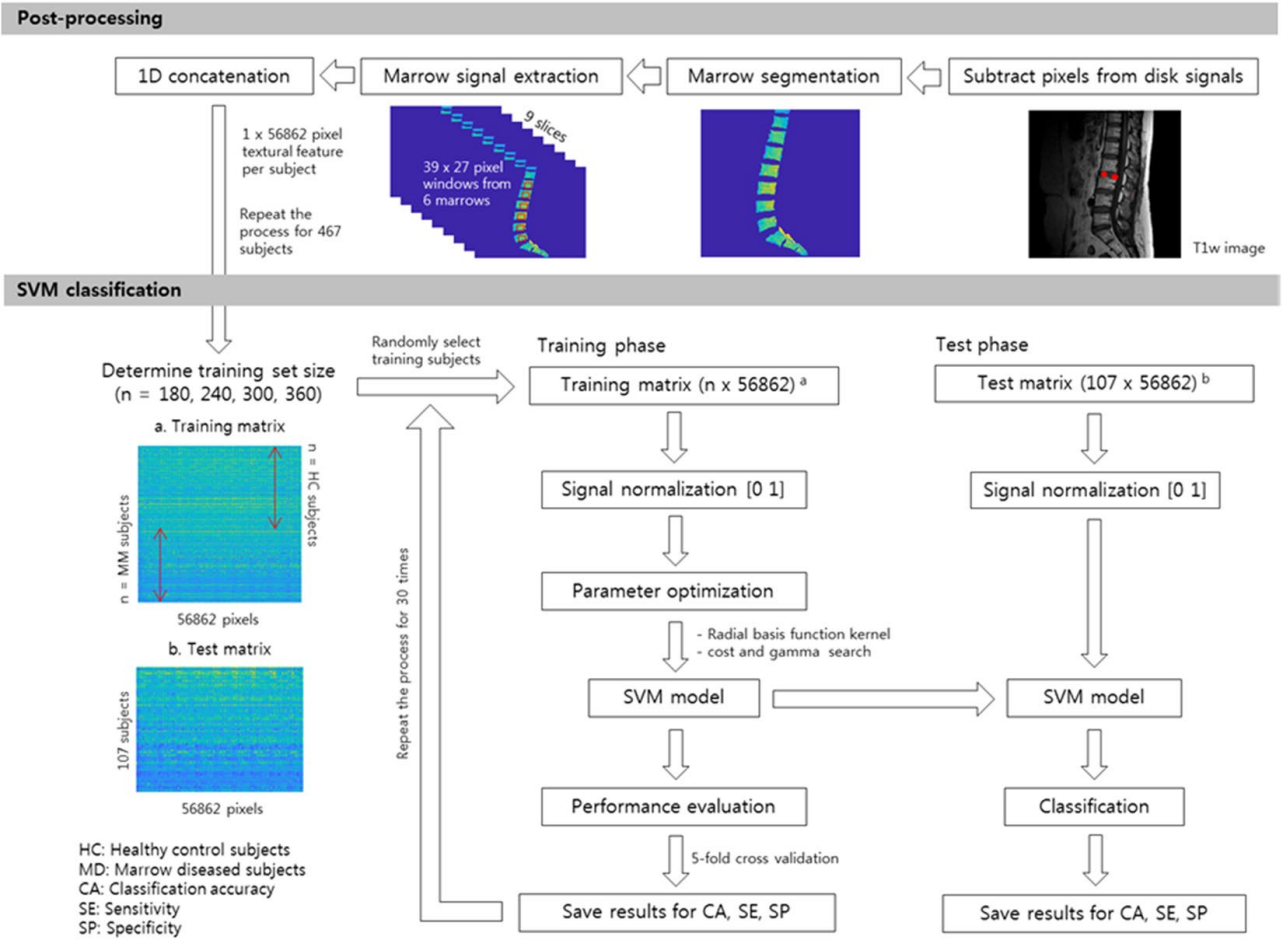
Flowchart illustrating the study process from postprocessing to training and test phase of SVM classification. The T1-weighted images were normalized by the annulus fibrosus of the non-degenerated intervertebral disks, and marrows were segmented using a 3-dimensional semi-automatic algorithm. The raw pixels from the segmented marrows were extracted and concatenated to a 1-dimensional vector. For SVM classification, the training matrix was formulated from randomly selected subjects, and the values within the matrix were normalized between 0 and 1. The kernel parameters were optimized to find the best SVM model, and the 5-fold cross validation was performed to estimate overall performance of the model. The final SVM model was applied to the test set to estimate accuracy, sensitivity and specificity.

The parameters used to construct a final SVM classifier model were experimented using LIBSVM version 3.21 (Library for Support Vector Machines, https://www.csie.ntu.edu.tw/~cjlin/libsvm/)^36^. Figure 5 summarizes the study process from data preparation to classification using SVM. A rectangular window was created to cover the marrow signals of the vertebral body from a single subject. Since each individual had different shapes and sizes, a minimal window size was selected to encompass the smallest marrow among the subject population. From a single slice, 6 windows were created from S1 to L1 vertebral bodies, and the same numbers of windows were selected from the three and nine consecutive slices, respectively. Lastly, the 2-dimensional window array was reshaped into a 1-dimensional vector, each of which was concatenated to a single textural feature per subject.

Once the most optimal kernel function and feature dimension were determined, the final SVM classifier model was experimented on the varying numbers of training sizes from 180 to 360. For each size of the training set, training was repeated 30 times, each with different combinations of the training sets randomly selected from the training population. The pixel values within the feature matrices were scaled from 0 to 1. The grid-search method was employed to find two kernel parameters, the cost function C and gamma, γ, which identifies the pair of C and γ with the best internal cross-validation accuracy. The 5-fold cross validation was performed to the training set to estimate overall performance of the model. A final SVM classifier model was generated to the entire training set using the optimized parameters.

### Comparison of predictive performances of SVM to human readers

The constructed SVM model was applied to the test set. Accuracy, sensitivity and specificity from the test set were regarded as the final outcomes. Two readers (S.K.L and J.Y.J with 3 and 10 years of experience in musculoskeletal radiology, respectively) blinded to clinical and laboratory results independently reviewed the test set. They determined the presence of hematologic diseases with five-level confidence scores: 0 = definitely absent, 1 = probably absent, 2 = equivocal, 3 = probably present, 4 = definitely present.

### Sample size estimation using a learning curve

A learning curve model was employed to estimate target classification accuracy at a given number of the training set size. The curve model is represented as an inverse power law function, where the classification accuracy is expressed as a function of a training set size given unknown coefficients of a, b and c. The learning curve is modeled by the following equation^1^:

1 $${\rm{y}}={\rm{f}}\,({\rm{x}};{\bf{a}},{\bf{b}},{\bf{c}})=(1-{\bf{a}})-{\bf{b}}{{\rm{x}}}^{{\bf{c}}}$$

where x is the training set size and y is the classification accuracy; a, b and c represent the minimum achievable error, learning rate and decay rate, respectively.

Using the observed classification accuracy at 13 different training sizes (0, 10, 20, 40, 60, 80, 100, 120, 140, 180, 240, 300, and 360), the unknown coefficients were estimated using a non-linear regression. In the MATLAB software, a function ‘fit’ was implemented using a Levenberg-Marquardt algorithm. The target sensitivity at the given training size was also estimated using the same equation.

### Statistical Analysis

The classification accuracy, sensitivity, specificity and diagnostic accuracy for training phases were estimated using an area under the receiver operating characteristic (ROC) curve (AUC)^37^. Sensitivity is the proportion of test positives among those who are truly diseased. Specificity is the proportion of test negatives among those who are not diseased. AUC is the measure of classification performance at various threshold settings, which tells the capability of the model in distinguishing different classes in the range of 0.5 and 1.

For the calculation of sensitivity, specificity, and interobserver agreements in human readers, 0–2 was regarded as negative, while 3–4 regarded as positive. Interobserver agreements (*к*) were calculated between SVM and readers. The *κ* values can be interpreted as poor (*κ* = 0), slight (*κ* = 0.0–0.2), fair (*κ* = 0.21–0.40), moderate (*κ* = 0.41–0.60), substantial (*κ* = 0.61–0.80), and almost perfect (*κ* = 0.81–1.00)^38^.

Sensitivity, specificity and accuracy between the SVM classifier and human readers were compared by McNemar statistics. AUCs were compared between the SVM classifier and two readers^39^. A multivariate logistic regression analysis was performed to estimate the influence of benign marrow signal changes including vertebral hemangioma, Modic type change, and fracture on classification results. P < 0.05 was considered significant for aforementioned statistics.

Finally, goodness-of-fit to the learning curve was evaluated using sum of squares due to error (SSE) and root mean squared error (RMSE). SSE measures the total deviation between the observed (*y*_*i*_) and predicted accuracies (*y*). The weight (*w*_*i*_) is the weighting applied to each data point and is usually *w*_*i*_ = 1 (Eq. 2). RMSE is the square root values of SSE divided by the residual degrees of freedom, which is defined as the number of data points (*n*) minus the number of fitted coefficients (*m*) (Eq. 3). SSE and RMSE values close to zero indicate a better fit. The curve-fitting and evaluation were separately performed for classification accuracy and sensitivity.

2 $${\rm{SSE}}=\,\sum _{i=1}^{n}wi{(yi-y)}^{2}$$

3 $${\rm{RMSE}}=\sqrt{\frac{SSE}{(n-m)}}$$

## Supplementary information

Supplementary table and figure
