# Supplementary material for: Machine Learning for Diagnosis of Hematologic Diseases in Magnetic Resonance Imaging of Lumbar Spines
Source: Sci Rep. 2019 Apr 15;9:6046. doi: 10.1038/s41598-019-42579-y (PMC6465258; doi:10.1038/s41598-019-42579-y)
Supplement: Supplementary file 1 — Supplementary table and figure [file 41598_2019_42579_MOESM1_ESM.pdf]

Machine Learning for Diagnosis of Hematologic Diseases in Magnetic Resonance Imaging of Lumbar Spines

Eo-Jin Hwang[1], M.S., *Joon-Yong Jung[1], M.D., Seul Ki Lee[1], M.D., Sung-Eun Lee[2], M.D., Won-Hee Jee[1], M.D.

Supplementary Table S1. Results of non-linear regression to the learning curve model and evaluation of goodness-of-fit

	Coefficients [95% Confidence interval]			Goodness-of-fit	
	a (minimum achievable error)	b (learning rate)	c (decay rate)	SSE	RMSE
Accuracy	0.999[0.974,1.03]	-0.576[-0.613,-0.539]	0.0616[0.0516,0.0716]	0.00131	0.0115
Sensitivity	1.001 [0.943,1.06]	-0.574[-0.653,-0.494]	0.0736[0.0519,0.0952]	0.00667	0.0258

Abbreviations: SSE = sum of squares due to error, RMSE = root means squared error.

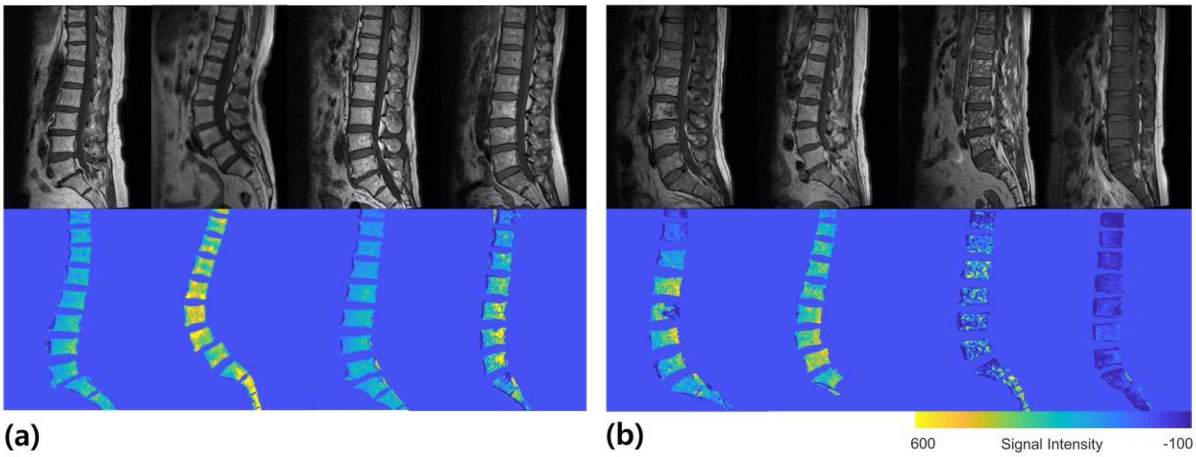

Supplementary Figure S1. Representative slices of the sagittal, T1-weighted images of the spines of the (a) normal controls and (b) patients with hematologic diseases (top), and the corresponding segmented and disk-normalized marrow images, which were obtained by subtracting the whole image from the annulus fibrosus of non-degenerated intervertebral disks and by segmenting marrows using a 3-dimensional semi-automatic algorithm (bottom).
